# Overview of three influenza seasons in Georgia, 2014–2017

**DOI:** 10.1371/journal.pone.0201207

**Published:** 2018-07-27

**Authors:** Ann Machablishvili, Giorgi Chakhunashvili, Khatuna Zakhashvili, Irakli Karseladze, Olgha Tarkhan-Mouravi, Mari Gavashelidze, Tamar Jashiashvili, Lela Sabadze, Paata Imnadze, Rodney S. Daniels, Burcu Ermetal, John W. McCauley

**Affiliations:** 1 National Center for Disease Control and Public Health, Tbilisi, Georgia; 2 Ivane Javakhishvili Tbilisi State University, Tbilisi, Georgia; 3 Worldwide Influenza Centre (WHO Collaborating Centre for Reference and Research on Influenza), The Francis Crick Institute, London, United Kingdom; St. Jude Children's Research Hospital, UNITED STATES

## Abstract

**Background:**

Influenza epidemiological and virologic data from Georgia are limited. We aimed to present Influenza Like Illness (ILI) and Severe Acute Respiratory Infection (SARI) surveillance data and characterize influenza viruses circulating in the country over three influenza seasons.

**Methods:**

We analyzed sentinel site ILI and SARI data for the 2014–2017 seasons in Georgia. Patients’ samples were screened by real-time RT-PCR and influenza viruses isolated were characterized antigenically by haemagglutination inhibition assay and genetically by sequencing of HA and NA genes.

**Results:**

32% (397/1248) of ILI and 29% (581/1997) of SARI patients tested were positive for influenza viruses. In 2014–2015 the median week of influenza detection was week 7/2015 with B/Yamagata lineage viruses dominating (79%); in 2015–2016—week 5/2016 was the median with A/H1N1pdm09 viruses prevailing (83%); and in 2016–2017 a bimodal distribution of influenza activity was observed—the first wave was caused by A/H3N2 (55%) with median week 51/2016 and the second by B/Victoria lineage viruses (45%) with median week 9/2017. For ILI, influenza virus detection was highest in children aged 5–14 years while for SARI patients most were aged >15 years and 27 (4.6%) of 581 SARI cases died during the three seasons. Persons aged 30–64 years had the highest risk of fatal outcome, notably those infected with A/H1N1pdm09 (OR 11.41, CI 3.94–33.04, p<0.001). A/H1N1pdm09 viruses analyzed by gene sequencing fell into genetic groups 6B and 6B.1; A/H3N2 viruses belonged to genetic subclades 3C.3b, 3C.3a, 3C.2a and 3C.2a1; B/Yamagata lineage viruses were of clade 3 and B/Victoria lineage viruses fell in clade1A.

**Conclusion:**

In Georgia influenza virus activity occurred mainly from December through March in all seasons, with varying peak weeks and predominating viruses. Around one third of ILI/ SARI cases were associated with influenza caused by antigenically and genetically distinct influenza viruses over the course of the three seasons.

## Introduction

Influenza viruses affect people of all ages and cause mild to severe disease sometimes leading to fatal outcomes [1–4]. Surveillance of Influenza Like Illness (ILI) and Severe Acute Respiratory Infection (SARI) represent an important tool for tracking trends of virus spread and changes in globally circulating influenza viruses [5]. The first pandemic of the 21^st^ century caused by influenza virus A/H1N1pdm09 demonstrated the importance of influenza surveillance worldwide. The monitoring of changes in seasonal influenza viruses plays a critical role in defining influenza vaccine composition [6, 7]. Moreover, data obtained from surveillance systems enables healthcare authorities to better understand timings of influenza activity and consequent mobilization of resources (e.g. vaccines, antivirals), notably prioritized vaccination of risk groups among other measures. However, limited data are available on influenza morbidity and mortality in Georgia [8–10] and none of these publications describe antigenic and genetic characteristics of seasonal influenza viruses circulating in the country.

Georgia is located in the Caucasus region and covers a territory of 69700 km^2^ with a population of around 3.7 million people. Prior to 2006, a nationwide population-based surveillance system for influenza and upper respiratory tract infection provided influenza data based only on clinical diagnosis without laboratory confirmation. In 2007, in collaboration with US CDC and other international stakeholders, sentinel surveillance of ILI and SARI was initiated. The National Influenza Center (NIC) at the National Center for Disease Control and Public Health, Georgia (NCDC&PH) now screens specimens collected at ILI/SARI surveillance sites all year round to monitor activity of influenza viruses.

In this study we describe epidemiological and virologic data for three consecutive influenza seasons obtained from ILI/SARI surveillance systems. The objectives were to: (1) define periods of influenza activity in Georgia; (2) assess the proportions of influenza infections among ILI and SARI cases; (3) determine most affected age groups; (4) describe epidemiological characteristics of influenza-associated fatal cases; and (5) determine antigenic and genetic profiles of influenza viruses circulating in the country.

## Materials and methods

### Setting and study design

ILI surveillance was carried out in one outpatient clinic with a predetermined catchment of approximately 60,000 residents in the capital city Tbilisi (5% of 1.2 million population). The Children’s Central Hospital, the largest children’s clinic of the country also in Tbilisi, and four hospitals in Kutaisi (the biggest city in Western Georgia) were selected as SARI surveillance sites (S1 Fig). Medical staff and epidemiologists at each site were trained on case definitions, specimen collection, storage and transportation, completion of individual patient questionnaires and ILI/SARI aggregated forms.

### Definition of ILI/SARI cases

The case definitions were standardized for all clinics involved in influenza sentinel surveillance using the 2014 revision of WHO case definitions [5]. ILI was defined as measured fever of ≥38°C and cough, with onset within the last 10 days. In addition, the SARI case definition implied the requirement for hospitalization.

### Sample and data collection

A systematic alternate day sampling method was used at the ILI and SARI surveillance sites [5]. Both oral and nasal swabs were taken from each patient and placed into a cryovial containing 1.5ml virus transport medium (VTM) and labeled with the patient’s ID number. Samples were stored at +4°C if transported to the NIC within 2–3 days, otherwise they were stored at -80°C. Specimens were kept at -80°C prior to molecular characterization if not tested immediately after reception at the NIC. Individual patient questionnaires (including demographics, clinical signs, vaccination and antiviral treatment status) were completed prior to sampling and more detailed questionnaires (including treatment, chronic underlying conditions and complications) were completed for fatal SARI cases. Trained clinicians were responsible for completing the questionnaires and sending aggregated forms on ILI/SARI referral, at the end of each week, to NCDC&PH epidemiologists involved in the study. These forms included for ILI the number visits by age groups within the catchment population and for SARI the number of cases, relative to total hospitalizations, by age group.

### Laboratory investigations

#### Influenza virus detection

Viral RNA was extracted with QIAamp Viral RNA Mini Kits (Qiagen, Germany) according to manufacturer’s instructions. Real-time RT-PCR was performed for influenza virus detection, influenza A subtyping and influenza B lineage determination (the latter being implemented during the 2015–2016 season). Specimens were screened for influenza viruses using molecular kits, positive controls and protocols provided by the US CDC [11]. Laboratory results were recorded as influenza negative or positive for seasonal influenza viruses (A/H1N1pdm09, A/H3N2, B, B/Victoria or B/Yamagata).

#### Virus isolation and antigenic characterization

Real Time RT-PCR positive specimens with Ct values <30 were inoculated onto Madin Darby Canine Kidney (MDCK) cells, incubated at 34°C and observed daily for 7 days for cytopathic effect. Haemagglutination assay was performed for confirmation of virus growth in cell culture using a 0.5% suspension of turkey red blood cells (RBCs). Preliminary antigenic characterization of influenza isolates was carried out by haemagglutination inhibition (HI) assay using sheep or goat antisera provided by the CDC, Atlanta in accordance with the WHO Manual [12]. For detailed antigenic analysis representative viruses from Georgia were sent to WHO-CCs in London and Atlanta where isolates were studied by HI using panels of post-infection ferret antisera raised against reference viruses.

#### Genetic characterization

Sequence analysis of hemagglutinin (HA) and neuraminidase (NA) genes of influenza viruses was performed by NIC, Georgia; Crick Worldwide Influenza Centre, London, UK, and CDC, Atlanta, USA. A/H3N2 and A/H1N1pdm09 specific primers and the Sanger sequencing protocol were provided to NIC Georgia by Walter Reed Army Research Institute (WRAIR), USA. cDNAs were subjected to BigDye^®^ terminator sequencing amplifications (Applied Biosystems) and the purified BigDye^®^ terminator products were subsequently sequenced using an ABI 3130XL Analyser. DNA sequencing data were processed and assembled using Sequencher 5.0 software (Gene Codes Inc., Ann Arbor, Michigan). Gene sequences of viruses from Georgia generated by Crick Worldwide Influenza Centre and CDC, and influenza viruses from various countries, were downloaded from the EpiFlu database of the Global Initiative on Sharing All Influenza Data (GISAID) (http://www.gisaid.org). Nucleotide and deduced amino acid sequences were aligned and analyzed using BioEdit (http://www.mbio.ncsu.edu/BioEdit/bioedit.html) and maximum likelihood phylogenetic trees were estimated using RaxML v8.2X with a GTRGAMMA substitution model (https://sco.h-its.org/exelixis/software.html), followed by annotation with amino acid substitutions defining nodes and individual virus gene products using treesub (https://github.com/tamuri/treesub/blob/master/README.md). Phylogenies were based on full-length open-reading-frames with the signal peptide component being removed from HA genes and stop codons removed from all genes: H1-HA 1647, H3-HA 1650, N1-NA 1407, N2-NA 1407, B/Vic-HA 1710, B/Yam-HA 1707, B/Vic-Na 1398 and B/Yam-NA 1398 nucleotides, respectively. Trees were visualized using FigTree (http://tree.bio.ed.ac.uk/software/figtree/) and highlighted using Adobe Illustrator CC 2015.3 (http://www.adobe.com/uk/products/illustrator/features.html). Sequence accession numbers for all reference and test viruses are given in S1 Table.

### Ethical considerations

Written informed consent was not required for this study. Verbal consent from patients and guardians in the case of children was sufficient for sample and data collection as the study was carried out under Georgia’s routine public health surveillance practice.

### Statistical analysis

Fisher’s exact test was used for statistical analysis of data with chi squared estimation of significance. P values of ≤0.05 were considered as statistically significant. Data analysis was performed using Epi Info (version 7). Median, range, and interquartile range (IQR) were calculated to assess continuous variables. As Georgia is in the Northern Hemisphere, an influenza season is defined as the time period from week 40 of each year to week 20 of the following year, when the vast majority of influenza detections are made, and only data for these periods were analyzed. ILI consultation rates per 100 000 population and SARI vs total hospital admissions were used for evaluating season dynamics. The seasonal threshold was calculated using WHO guidelines and methodology [5].

## Results

### Influenza activity in Georgia

A total of 12,912 ILI and 10,218 SARI patients met the ILI/SARI case definitions at sentinel sites during three consecutive influenza seasons 2014–2017; of these, 1,248 (9.7%) ILI and 1,997 (19.5%) SARI patients were sampled and tested for influenza. Overall 30% (978/3,245) specimens were positive: 397 (32%) ILI and 581 (29%) SARI (including four specimens: three ILI and one SARI—positive for both influenza A and B viruses).

**Fig 1:**
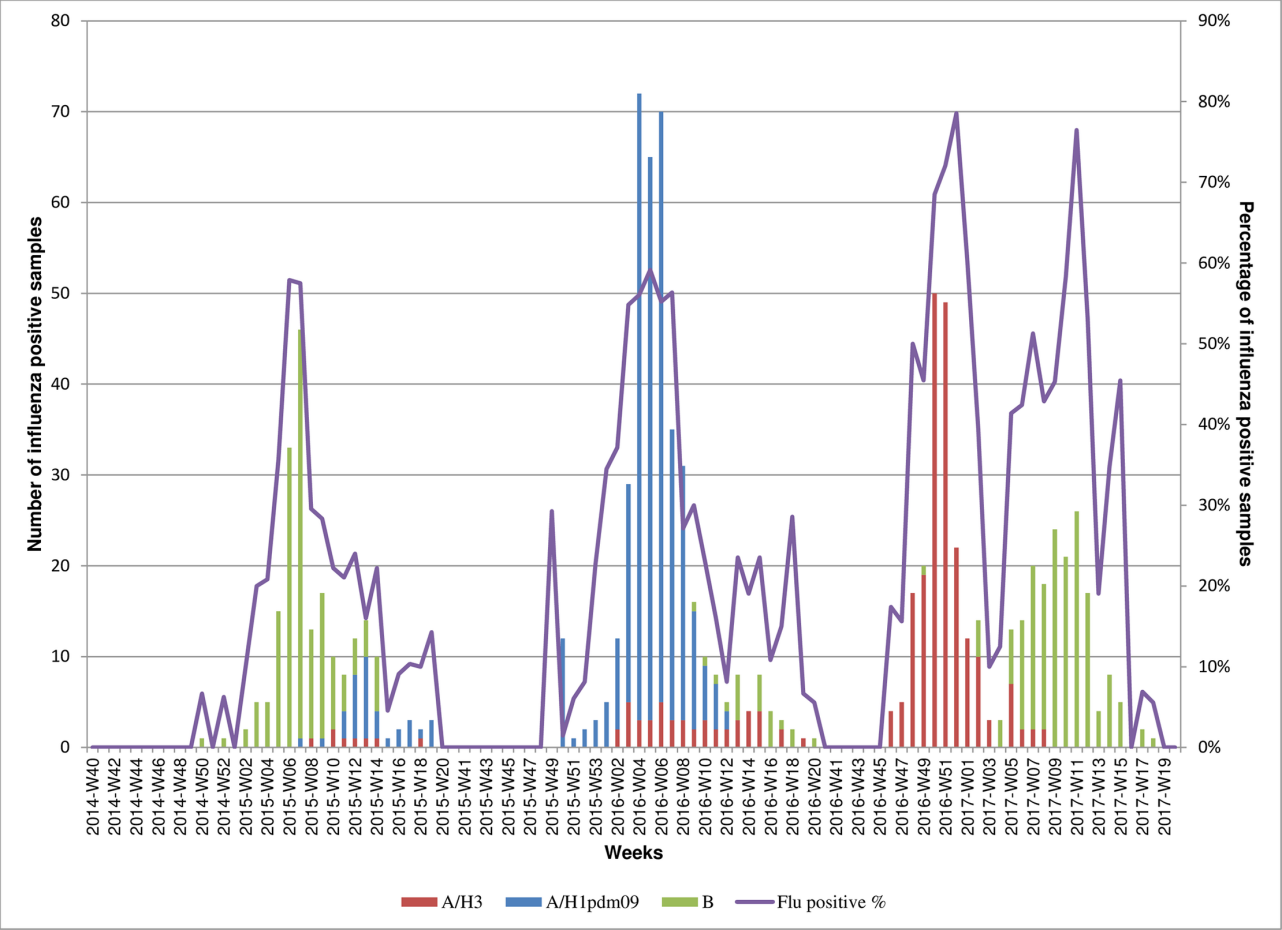
Distribution of influenza positive specimens by week during consecutive influenza seasons, 2014–2017, Georgia. Numbers of influenza positive samples are displayed in vertical bars color-coded to show various types/subtypes of influenza viruses. The percentage of all samples collected each week that tested positive for influenza viruses is shown by the line.

Influenza detection median weeks and prevailing viruses varied between seasons: in 2014–2015 the median week of total influenza detections was week 7/2015 (IQR weeks 6–9) with influenza B viruses dominating (79%); in 2015–2016 the median week of total influenza detections was week 5/2016 (IQR weeks 3–7) with A/H1N1pdm09 viruses dominating (83%); and the 2016–2017 season showed a bimodal distribution of influenza detections—the first wave was caused by A/H3N2 (55%) with a median week of 51/2016 (IQR weeks 50–52) and the second by influenza B (45%) with a median week of detections of 9/2017 (IQR weeks 7–11) (Fig 1). Of the 161 influenza B positive specimens from the 2014–2015 season 12 (7.5%) selected at random were sent to WHO CC London where all were characterized as B/Yamagata lineage viruses. Introduction of a B-lineage determining real time RT-PCR assay in the fall of 2015 showed all influenza B viruses of the 2015–2016 and 2016–2017 seasons tested at NIC, Georgia were of the B/Victoria lineage.

### ILI surveillance

**Fig 2:**
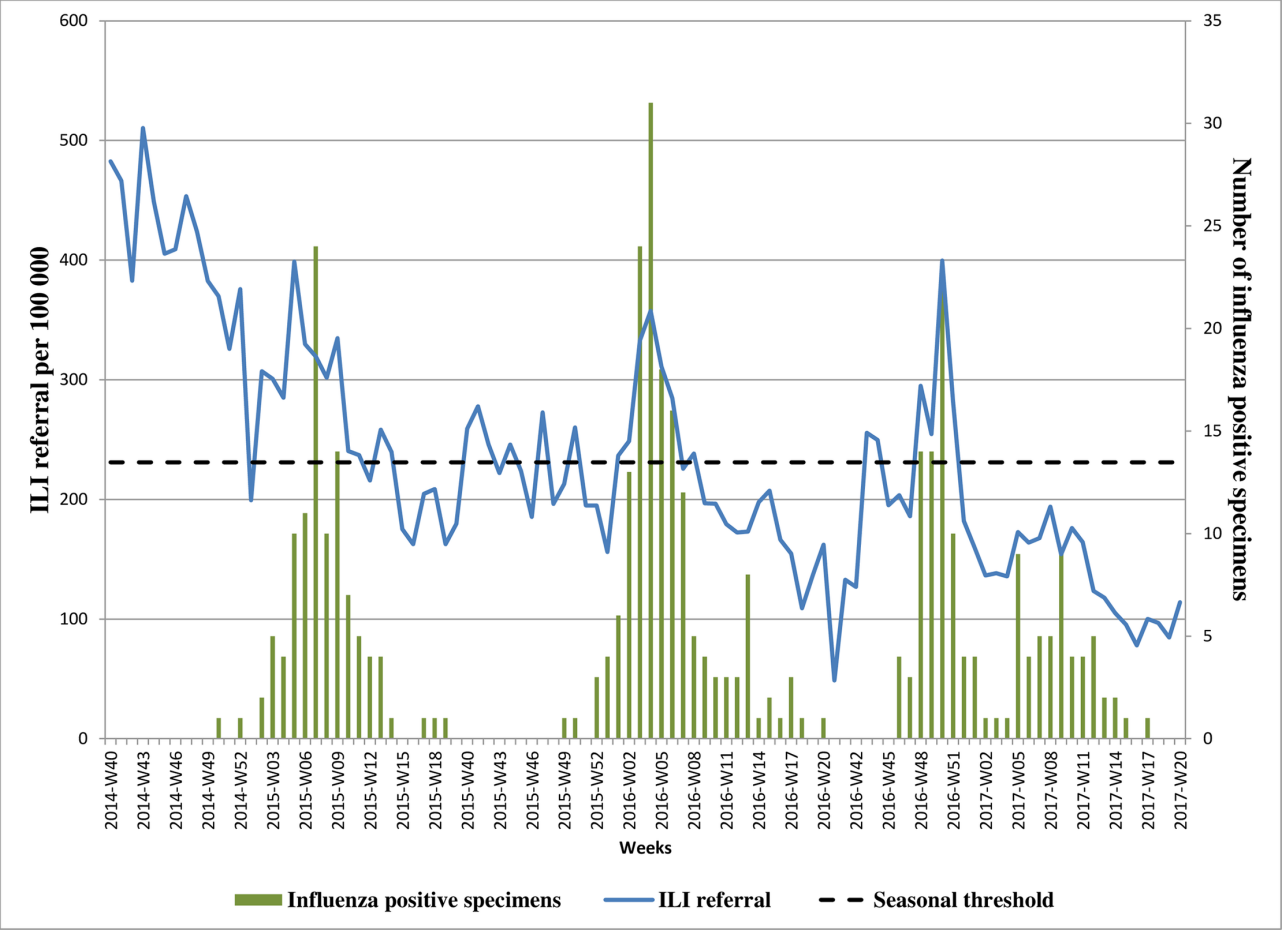
Referral of ILI cases to outpatient clinic vs laboratory-confirmed influenza detections by week, 2014–2017, Georgia. Vertical bars represent numbers of influenza positive samples among ILI cases each week over three consecutive seasons. The blue line shows weekly referrals of ILI cases to the outpatient clinic per 100 000 population.

Of the 397 influenza virus-positive ILI samples over three seasons (2014–2017) 132 (33%) were A/H1N1pdm09, 121 (30%) were A/H3N2 and 147 (37%), influenza B (three were positive for both influenza A and B viruses). During influenza activity periods weekly outpatient visits for ILI rose in parallel with laboratory-confirmed influenza virus detection (Fig 2). Based on our data seasonal threshold was determined as an ILI referral of 231/100,000. The 2014–2015 influenza season had an unusually high referral of ILI cases which was considerably earlier than laboratory-confirmed influenza virus detection, presumably due to circulation of other respiratory viruses. ILI referral exceeded the threshold from week 40/2014 until week 14/2015. In 2015–2016 ILI visits exceeded the threshold from week 50/2015 to week 8/2016. For 2016–2017, in spite of the bimodal distribution of influenza circulation and a longer season, ILI referral exceeded the threshold for only weeks 43-51/2016 during the period of elevated A/H3N2 activity.

Overall influenza detection was highest 48% (133/276) among children aged 5–14 years (OR = 2.51, CI 1.90–3.30, p<0.001) (Table 1). Children under 1 year showed the lowest level of influenza virus positivity (17%, 11/63; OR = 0.36, CI 0.19–0.69, p = 0.001) and cases under 5 years of age showed lower levels of influenza infection compared to older patients (25.7%, OR = 0.64, CI 0.49–0.83, p = 0.001). Stratification of positive cases based on dominant influenza subtypes for particular seasons revealed that children aged 5–14 years were the group most affected by influenza B viruses (2014–2015 season—40%, OR = 2.95, CI 1.75–4.96, p<0.001; 2016–2017 season—27%, OR = 4.10, CI 2.17–7.73, p<0.001) followed by the age group 15–29 years (2016–2017 season, 24%, OR = 2.45, CI 1.20–5.03, p = 0.012). A/H3N2 viruses were mostly seen in children aged 5–14 years (2016–2017 season, 35%, OR = 2.22, CI 1.31–3.77, p = 0.004) while the highest A/H1N1pdm09 virus detection rate was among the 30–64 year-olds (2015–2016 season, 32%, OR = 1.76, CI 1.13–2.75, p = 0.012).

**Table 1:** Age distribution of influenza confirmed ILI and SARI cases, 2014–2017 seasons, Georgia.

	Both surveillance systems		SARI surveillance system		ILI surveillance system	
	Total count	Influenza positive count (%)	Total count	Influenza positive count (%)	Total count	Influenza positive count (%)
Overall	3245*	978 (30.1%)	1997	581 (29.1)	1248	397 (31.8)
Age						
<1 year	614	88 (14.3)	551	77 (14.0)	63	11 (17.5)
1–4 years	1022	269 (26.3)	638	165 (25.9)	384	104 (27.1)
5–14 years	600	240 (40.0)	324	107 (33.0)	276	133 (48.2)
15–29 years	325	126 (38.8)	141	75 (53.2)	184	51 (27.7)
30–64 years	486	192 (39.5)	197	109 (55.3)	289	83 (28.7)
≥65 years	188	60 (31.9)	143	48 (33.6)	45	12 (26.7)

For each age group total numbers of ILI and SARI specimens tested for influenza viruses are shown with numbers positive given in the adjacent column and % positivity shown in parentheses.

* Ages of 7 ILI and 3 SARI case patients were unknown

Only 18 of 1248 swabbed ILI participants had been vaccinated during the three seasons; of these, eight persons became ill with influenza A/H3N2 and two with A/H1N1pdm09.

### SARI surveillance

**Fig 3:**
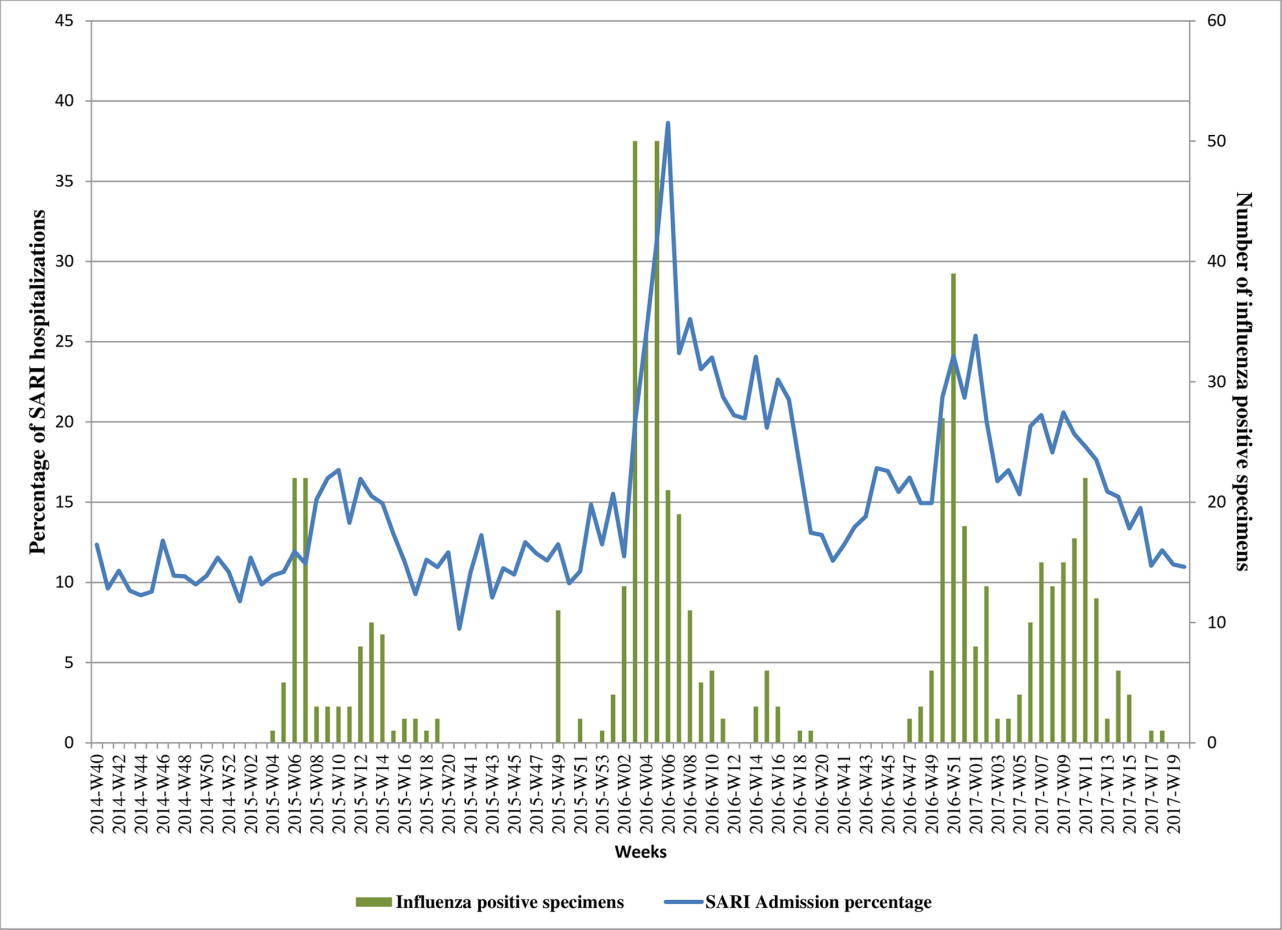
Admission of SARI cases to hospitals vs laboratory-confirmed influenza detections by week, 2014–2017, Georgia. Vertical bars show numbers of influenza positive specimens among SARI cases each week. The blue line represents percentages of influenza-positive cases among total SARI hospital admissions on a weekly basis.

Out of 581 specimens testing positive for influenza viruses (Table 1), 241 (41%) were A/H1N1pdm09, 138 (24%) A/H3N2 and 203 (35%) influenza B (one was positive for both influenza A/H3N2 and B). During weeks when influenza activity was high (Fig 1) SARI admissions rose above an approximate 10% threshold: in 2014–2015 SARI cases made up between 10% and 17% of weekly hospital admissions; in 2015–2016 and 2016–2017 seasons these rates were higher, ranging from 11% to 39% and from 11% to 25%, respectively (Fig 3).

Influenza virus detection rates among hospitalized patients were highest, almost equally, in the age groups 30–64 years (55%, OR = 3.48, CI 2.58–4.69, p<0.001) and 15–29 years (53%, OR = 3.03, CI 2.14–4.28, p<0.001) (Table 1). Patients under 1 year were the least affected age group by influenza resulting in SARI (14%, OR = 0.30, CI O.23-0.39, p<0.001) and children <5 years showed a lower positivity rate compared to older patients (25.9%, OR = 0.35, CI O.29-0.43, p<0.001). Stratified analysis of confirmed cases according to dominant influenza virus by season showed that influenza B related hospitalizations was highest in age groups 15–29 years (2014–2015 season, 30%, OR = 3.19, CI 1.34–7.59, p = 0.013; 2016–2017 season, 46%, OR = 3.98, CI 2.26–7.01, p<0.001) and 5–14 years (2016–2017 season, 33%, OR = 2.30, CI 1.40–3.80, p = 0.001). A/H3N2 virus detection was mostly seen in cases aged 30–64 years (season 2016–2017, 35%, OR = 2.39, CI 1.36–4.20, p = 0.002); and severe cases associated with A/H1N1pdm09 viruses in 2015–2016 were most frequently observed in patients aged 30–64 years (59%, OR = 5.04, CI 3.12–8.16, pP<0.001) and 15–29 years (49%, OR = 2.99, CI 1.74–5.16, p<0.001).

Among 1,997 sampled SARI cases, only 25 were vaccinated against influenza; of these, three tested positive for A/H1N1pdm09 and three for A/H3N2.

### Influenza-associated fatal cases

During the three influenza seasons, 27 (4.6%) patients out of 581 laboratory-confirmed influenza SARI cases died, all of whom were patients in Kutaisi SARI sentinel hospitals. Of the fatal cases 19 (70%) were male and 8 (30%) female. Influenza A/H1N1pdm09 was associated with 20 (74.1%), A/H3N2 five (18.5%) and influenza B two (7.4%) deaths (S2 Table). Thirteen (48%) of 27 deaths occurred during the 2015–2016 season when the dominant virus was A/H1N1pdm09 and, despite the dominance of influenza B viruses in 2014–2015, there were still seven deaths associated with A/H1N1pdm09 that season. Patients infected with A/H1N1pdm09 had a higher risk of fatal outcome compared to A/H3N2 and influenza B infection (OR 4.31, CI 1.75–10.35, p = 0.001). The age of patients who died ranged from 1 to 84 years (median 55 years, IQR 52–64), with 18 (67%) of 27 fatal cases being in the 30–64 years age group, yielding a high odds of fatal outcome (OR 10.18, CI 4.43–23.36 <0.001). A/H1N1pdm09 influenza confirmed cases aged 30–64 years had statistically significant higher risk for a fatal outcome compared to those in other age groups (OR 11.41, CI 3.94–33.04, p<0.001,). Twenty-five (93%) of 27 lethal cases had at least one underlying condition: the leading comorbidities were cardiovascular (12/27; 44%) and neurological disorders (7/27; 26%). The median time between symptom-onset and hospitalization was 4 days (range 0–12 days, IQR 3–6 days). Oseltamivir was administered to all patients except one; however, only three patients received antiviral treatment within 48 hours after the onset of clinical signs, the median time between disease onset and antiviral prescription was 6 days (range 1–13, IQR 4–7 days). The median time between symptomatic illness onset and influenza-associated death was 6 days (range 3–44, IQR 1–13 days). Only one patient was vaccinated against influenza, a 73 year old male resident of a disabled persons center suffering with mental and kidney health problems, who subsequently died.

### Antigenic and genetic characterization of influenza viruses

#### A/H1N1pdm09

**Fig 4:**
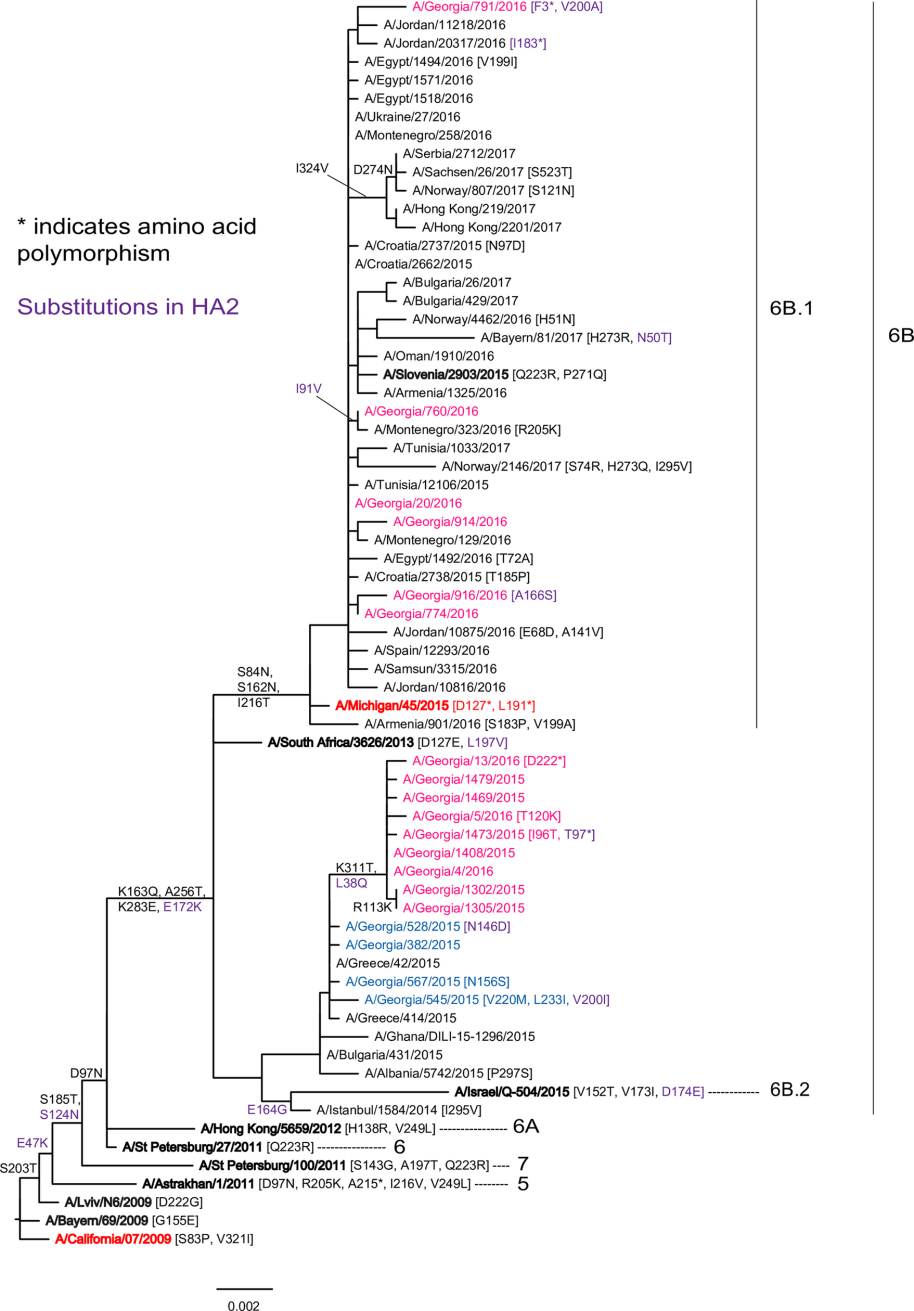
HA gene phylogeny of influenza A/H1N1pdm09 viruses detected in Georgia during 2014–2015 and 2015–2016 seasons. Vaccine viruses are indicated in red, viruses from Georgia for 2014–2015 and 2015–2016 seasons are shown in blue and pink respectively. Reference and vaccine viruses against which post-infection ferret antisera were raised for use in HI assays are in bold type. The scale bar represents nucleotide substitutions per site.

Eighteen (86%) of 21 viruses were successfully recovered by WHO CC London and studied by HI assay. All but two viruses were well recognized in HI assays, within 2-fold of the homologous titer, by antiserum raised against the vaccine virus, A/California/7/2009; A/Georgia/567/2015 and A/Georgia/791/2016 were recognized at titers 4- and 16-fold lower respectively. Both these viruses also showed reduced recognition, compared to the respective homologous titers, by antisera raised against several reference viruses. While A/Georgia/567/2015 carried an HA1 substitution (N156S; Fig 4) in a position known to affect antigenicity [13] no such substitutions were observed in either the HA or NA of A/Georgia/791/2016.

Sequencing of HA and NA genes of 19 A/H1N1pdm09 viruses was performed (Fig 4, S2 Fig). All four 2014–2015 season viruses belonged to the 6B genetic group characterized by encoding the amino acid substitutions P83S, D97N, K163Q, S185T, S203T, A256T, K283E and I321V in HA1 and E47K, S124N and E172K in HA2 compared to A/California/7/2009 and clustered with the reference virus A/South Africa/3626/2013. The NA of these four viruses differed from A/California/7/2009 by thirteen amino acid substitutions: I34V, L40I, N44S (gain of a glycosylation site), I117M, N200S, V241I, N248D, I321V, Y351F, I365T, N369K, N386K (loss of a glycosylation site) and K432E.

HA genes of nine viruses circulating early in the 2015–2016 season belonged to group 6B and encoded two additional amino acid substitutions K311T in HA1 and L37Q in HA2. The NA sequences of these viruses also fell in genetic group 6B and carried additional substitutions: T289I and I396M. The HA genes of six viruses from later in the 2015–2016 season fell into genetic subgroup 6B.1 characterized by encoding the substitutions S84N, S162N (introducing a new potential glycosylation site) and I216T in HA1 and clustered with reference virus A/Slovenia/2903/2015. NA genes of the same viruses also belonged to subgroup 6B.1, encoding the substitutions V13I, K264I, N270K and I314M in the NA glycoprotein.

#### A/H3N2

A/H3N2 viruses have undergone extensive evolution in recent years and a number of genetic clades/groups have been designated, indicated below in parentheses after virus names. Of 28 A/H3N2 viruses from the three seasons 22 (79%) were recovered by the WHO CCs.

In the 2014–2015 season all four A/H3N2 viruses were cultured from clinical samples but only one virus, A/Georgia/532/2015, had HA activity. It was poorly recognized by antisera raised against the 2014–2015 season vaccine virus, egg-propagated A/Texas/50/2012 (3C.1), and antisera raised against both cell culture-propagated and egg-propagated cultivars of the 2015–2016 season A/Switzerland/9715293/2013 (3C.3a) vaccine virus. However, A/Georgia/532/2015 was recognized well by antisera raised against cell culture-propagated A/Victoria/361/2011(3C.1) and both cell culture-propagated and egg-propagated cultivars of A/Stockholm/6/2014 (3C.3a).

Five of ten viruses from the 2015–2016 season were recovered, based on detection of sialidase activity, but only three had HA activity. Antiserum raised against the 2015–2016 season vaccine virus, egg-propagated A/Switzerland/9715293/2013 (3C.3a), recognized all three viruses poorly, but they were recognized by antiserum raised against the 2016–2017 season vaccine virus, egg-propagated A/Hong Kong/4801/2014 (3C.2a), at titers only 4-fold lower than the homologous titer of the antiserum.

Twelve viruses were isolated from 14 samples of the 2016–2017 season, but 11 were unable to agglutinate RBCs and only one virus (A/Georgia/1819/2016) was analyzed by HI assay. It was recognized by an antiserum raised against the recommended 2017–2018 season vaccine virus, egg-propagated A/Hong Kong/4801/2014, at a titer within 4-fold of the homologous titer of the antiserum.

**Fig 5:**
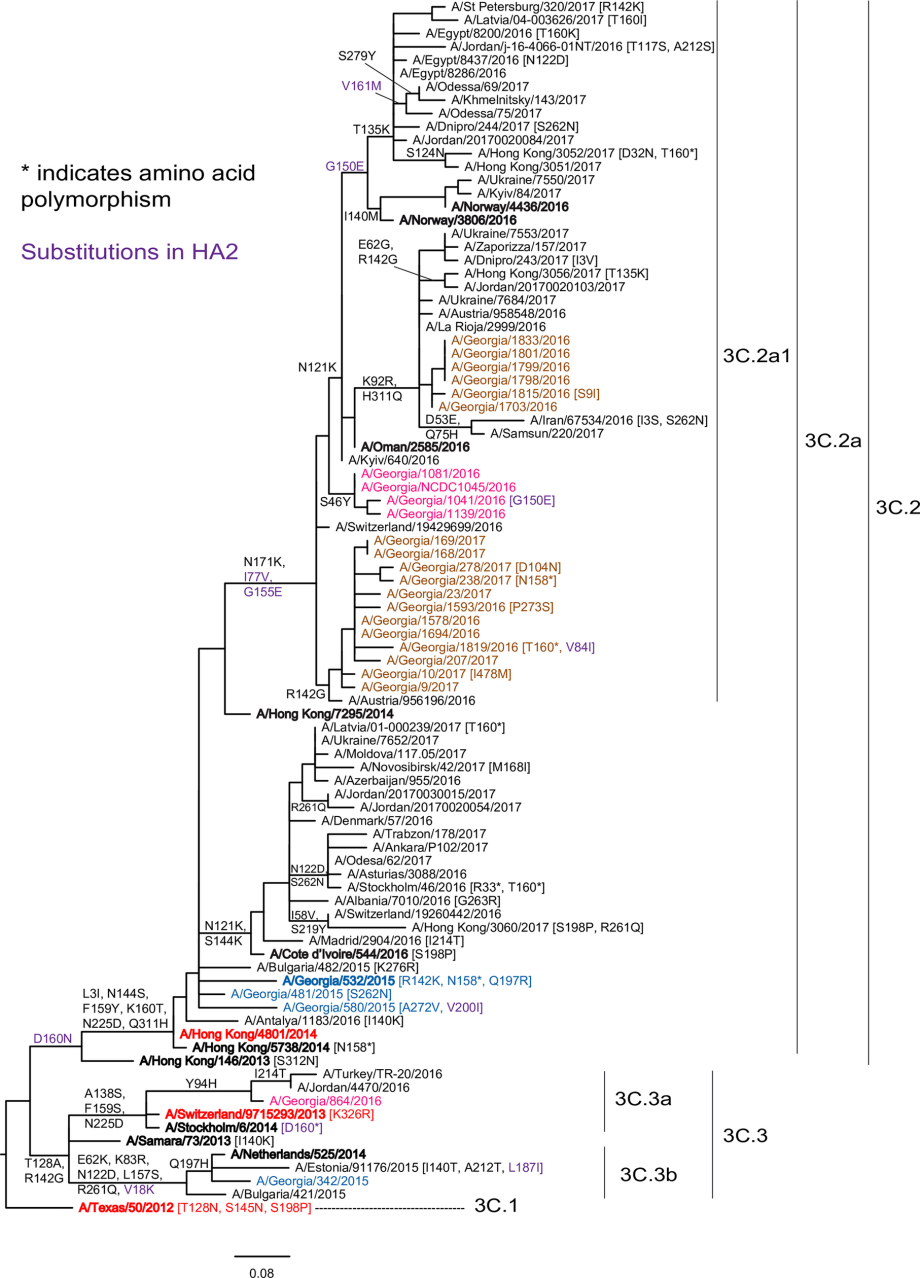
HA gene phylogeny of influenza A/H3N2 viruses detected in Georgia during three influenza seasons 2014–2017. Vaccine viruses are indicated in red; 2014–2015, 2015–2016 and 2016–2017 viruses from Georgia in blue, pink and brown respectively. Reference and vaccine viruses against which post-infection ferret antisera were raised for use in HI assays are in bold type. The scale bar represents nucleotide substitutions per site.

In total, 27 A/H3N2 viruses were sequenced (Fig 5, S3 Fig). Analysis of HA and NA genes of four viruses from the 2014–2015 season revealed that one virus belonged to genetic subgroup 3C.3b, encoding HA amino acid substitutions compared to the 3C.3 group: E62K, K83R, N122D (losing a glycosylation site), L157S, R261Q in HA1 and V18K in HA2, with this virus (A/Georgia/342/2015) carrying HA1 Q197H, as did some other viruses of the 3C.3b genetic subgroup. The NA gene of A/Georgia/342/2015 carried mutations encoding amino acid substitutions I26T, M51V, V143G, E221D, V263I and T434N. The HA gene of the other three viruses clustered in subgroup 3C.2a, characterized by amino acid substitutions L3I, N144S (resulting in the loss of a potential glycosylation motif), F159Y, K160T (resulting in the gain of a potential glycosylation site), N225D and Q311H in HA1. A/Georgia/532/2015 (3C.2a) carried HA1 amino acid substitutions R142K and Q197R and showed polymorphism at the 158–160 glycosylation motif which, when glycosylated in typical 3C.2a viruses, prevents the agglutination of RBCs. The NA genes of the 3C.2a viruses clustered together and encoded amino acid substitutions T267K and I380V in the NA glycoprotein.

HA gene sequence analysis of 2015–2016 season viruses showed that four of the five fell in subclade 3C.2a1 and one in clade 3C.3a, the A/Switzerland/9715293/2013 clade. The HA gene of the 3C.3a virus encoded HA1 amino acid substitution Y94H, commonly seen in 3C.3a viruses collected during the 2015–2016 season. The NA gene of this virus clustered with other 3C.3a subclade viruses. 3C.2a1 viruses from Georgia had, in addition to the clade defining substitutions (N171K in HA1 and I77V and G155E in HA2), the HA1 amino acid substitution S46Y. The NA genes of these four viruses clustered with other 3C.2a1 subclade viruses and differed from the A/Switzerland/9715293/2013 vaccine virus by encoding the substitutions S245N (resulting in the gain of a new glycosylation motif), S247T (a substitution that maintained a glycosylation motif at residues 245–247), T267K, D339N, I380V, T392I and P468H.

All 18 sequenced viruses of the 2016–2017 season from Georgia belonged to subclade 3C.2a1. Two viruses showed mixed sequences encoding the glycosylation motif at residues 158–160 in HA1 following culture; one of them, A/Georgia/1819/2016, was able to agglutinate RBCs. The HA genes of viruses from Georgia clustered in two subgroups: the first encoding an HA1 R142G substitution (12 viruses) and the second with HA1 amino acid changes K92R, N121K and H311Q (6 viruses). The NA genes of viruses from Georgia had the same clustering pattern as the HA genes and the NAs of all differed from that of the A/Hong Kong/4801/2014 vaccine virus by 7 amino acid substitutions: S245N (resulting in the gain of a new glycosylation site), S247T (maintaining the glycosylation motif), I231V, T267K, I380V, T392I and P468H. The NAs of 12 viruses also carried the amino acid substitutions L140I, V143M, S315R and D339N.

#### Influenza B

**B/Yamagata lineage**

Eleven (92%) of twelve viruses of the 2014–2015 season were successfully recovered. In HI analyses all but two viruses showed reduced recognition by antiserum raised against the egg-propagated B/Massachusetts/02/2012 vaccine virus (clade 2) used in the 2014–2015 season; however, all test viruses were recognized very well by antisera raised against egg-propagated viruses from clade 3 (including B/Phuket/3073/2013, the recommended influenza B component for 2015–2016 trivalent vaccines).

Sequence analysis of eight viruses showed that HA and NA genes belonged to clade 3, the B/Wisconsin/1/2010 –B/Phuket/3073/2013 clade (S4 and S5 Figs). All viruses differed from the 2014–2015 season vaccine virus, B/Massachusetts/2/2012, by 11 HA1 amino acid substitutions (K48R, A108P, N116K, S150I, N165Y, L172Q, A181T, N202S, G229D, K298E and E312K) and by 13 NA amino acid substitutions (I45V, H65R, A68T, L73P, I106T, T125K, K186R, V248I, R295S, E320K, K343E, D463N and A465T). The HA and NA genes of 7 viruses formed a tight cluster with the NAs being defined by Q42R amino acid substitution (S5 Fig).

**B/Victoria lineage**

Seven (54%) of 13 viruses from the 2015–2016 season were recovered and analyzed by HI. Two viruses reacted well with post-infection ferret antiserum raised against egg-propagated B/Brisbane/60/2008, the recommended influenza B component in northern hemisphere quadrivalent vaccines. The other five were recognized poorly by the same antiserum, but were recognized well by antisera raised against two cell culture-propagated viruses (B/Ireland/3154/2016 and B/Nordrhein-Westfalen/1/2016) genetically closely related to, and serving as surrogates for, cell-propagated B/Brisbane/60/2008.

Eleven (92%) of 12 viruses from the 2016–2017 season were recovered. All but two were poorly recognized by antiserum raised against the vaccine virus egg-propagated B/Brisbane/60/2008 but recognized well by antisera raised against cell culture-propagated reference viruses (B/Hong Kong/514/2009, B/Ireland/3154/2016 and B/Nordrhein-Westfalen/1/2016) at titers generally similar to the respective homologous titers of the antisera, all of which were low.

HA and NA genes of the 18 viruses fell into clade 1A, the B/Brisbane/60/2008 vaccine virus clade, but carried amino acid substitutions compared to B/Brisbane/60/2008: I117V and N129D in HA1 and I120V, K220N, S295R, N340D, E358K and D384G in the NA. Nine of 11 viruses from the 2016–2017 season carried additional substitutions of I45M, T72A, P336S in the NA (S6 and S7 Figs).

## Discussion

We present an analysis of data obtained from ILI and SARI sentinel surveillance sites and virus characterization to provide an overview of three consecutive influenza seasons in Georgia. The majority of laboratory-confirmed influenza detections were seen from December through to March, but seasonal peaks occurred in different weeks and coincided with those observed in the European region [14–16]. Influenza virus dominance was similar to that in Europe but for the 2014–2015 season when influenza B predominated in Georgia and the Ukraine, A/H3N2 viruses predominated in other European countries or co-dominated with influenza B [17–20].

During a season approximately one third of both ILI and SARI cases sampled by sentinel sites in Georgia were associated with influenza virus infection. Increased ILI consultation rates at outpatient clinics correlated with raised numbers of laboratory-confirmed influenza infections. The same trend was observed for SARI hospital admissions: the proportion of SARI cases among total hospitalizations doubled during peak weeks of influenza activity compared to weeks with low or no influenza detections. The number of SARI hospitalizations was lowest in 2014–2015, an influenza B season, and highest in 2015–2016 when A/H1N1pdm09 viruses predominated.

Children under 5 years of age were influenza virus-positive less frequently compared to older patients, reflecting the reports of a recent Egyptian study [21]. In our study the influenza confirmation rate was highest among ILI patients aged 5–14 years while the proportions of influenza-associated SARI cases were highest in the age groups 15–29 and 30–64 years. Such an age distribution might be influenced by the fact that during weeks of no or low influenza activity referral and/or sampling of children, notably those under 5 years of age, was higher compared to older patients; the rate of influenza detections in children could be lowered by this increased referral compared to adults.

Among ILI patients those aged 5–14 years were most often infected with influenza A/H3N2 or B viruses, while A/H1N1pdm09 infection was most commonly observed in individuals aged 30–64 years; similar observations have been described in Bulgaria [22]. The proportion of SARI cases testing positive for influenza A/H3N2 was highest in the age group 30–64 years, influenza B related hospitalization was mainly observed in the age groups 5–14 and 15–29 years but the majority of viruses were not ascribed to a lineage, while A/H1N1pdm09 viruses were detected most frequently in adults aged 15–29 and 30–64 years.

Of influenza-confirmed SARI cases in Georgia approximately 5% died while the percentage of fatal outcomes among hospitalized influenza-confirmed cases has been reported to vary from 0 to 4% in other countries [21, 23–25]. A fatal outcome was predominantly associated with A/H1N1pdm09 infection and patients in the age group 30–64 years had the highest probability of a fatal outcome. Our data are consistent with findings from other countries showing that adults were at higher risk of death than younger patients when infected with A(H1N10pdm09 viruses [25–28]. The vast majority of fatal cases suffered with at least one chronic condition, with cardiovascular and neurological disorders being the leading comorbidities. In a previous study based on data collected during the 2009 pandemic and post-pandemic seasons in Georgia, the major risk factors for a fatal outcome were lung disease, heart disease and pregnancy [10].

Antigenic and genetic characterization of influenza viruses from Georgia mostly showed similarities to strains circulating worldwide. Since their emergence A/H1N1pdm09 viruses have evolved forming eight genetic groups [29]. Viruses of recent years mainly fall into genetic group 6 which has three sub-divisions 6A, 6B and 6C. As observed globally, Georgian viruses from 2014–2015 and the early part of 2015–2016 seasons belonged to the 6B clade while those later in the 2015–2016 season fell into subgroup 6B.1 [29]. The 6B and 6B.1 viruses carried several mutations in HA and NA genes compared to the A/California/7/2009 vaccine virus. Five HA amino acid substitutions were located in four antigenic sites (Sa, Sb, Ca1, Ca2) while substitution S185T (190 loop) and residue 222 polymorphism in one virus were in the vicinity of the receptor-binding site; such substitutions can affect the antigenic properties of A/H1N1pdm09 viruses [30]. However, the majority of A/H1N1pdm09 viruses from Georgia, and those detected worldwide, remained antigenically similar to the A/California/7/2009 vaccine virus as assessed with post-infection ferret antisera.

Over the period of this study A/H3N2 viruses evolved rapidly genetically and showed some antigenic change, antigenic drift, such that three vaccine viruses were recommended: A/Texas/50/2012-like (3C.1) for 2014–2015, A/Switzerland/9715293/2013-like (3C.3a) for 2015–2016 and A/Hong Kong/4801/2014-like (3C.2a) for 2016–2017 seasons respectively (http://www.who.int/influenza/vaccines/virus/recommendations/en/). In 2014–2015, viruses from Georgia fell in genetic clades 3C.2a and 3C.3b while the majority of A/H3N2 viruses detected in the northern hemisphere belonged to clades 3C.2a and 3C.3a and the vaccine was reported to have low effectiveness [31, 32]. Similar clade mismatching was observed in 2015–2016 (3C.2a1 and 3C.3a) and 2016–2017 (3C.2a1) in Georgia, with 3C.2a1 subclade viruses predominating in some other parts of the world during both seasons [33]. Worldwide multiple mutations were detected in A/H3N2 viruses with many encoding clade defining HA amino acid substitutions, some of which were located in antigenic sites A (7), B (7), D (2) and E (4) and/or altered HA glycosylation patterns; modifications known to cause antigenic drift [34, 35]. However, the great majority of 3C.3a, 3C.2a and 3C.2a1 genetic group viruses yielded poor/no agglutination of RBCs, making antigenic characterization by HI impossible. Based on those that could be analyzed by HI, viruses isolated in Georgia and worldwide over the 2014–2017 seasons clearly differed antigenically from A/Texas/50/2012, while viruses in 3C.3a, 3C.2a and 3C.2a1 genetic groups appeared more closely related when assessed using post-infection ferret antisera raised against multiple representatives of each group.

B/Yamagata viruses from Georgia in the 2014–2015 season were antigenically similar to B/Phuket/3073/2013-like viruses (clade 3), as approximately 70% of the influenza B/Yamagata lineage viruses circulating in the European region [17], and differed from the B/Massachusetts/02/2012 vaccine virus (clade 2). HA genes of all clade 3 viruses encoded single amino acid substitutions in each antigenic site: the 120-loop, the 150-loop, the 160-loop and the 190-helix compared to viruses falling in clade 2. Amino acid substitutions in the 120-loop are known to alter virus antigenicity [36], so this might explain poor recognition of clade 3 viruses by antiserum raised against the B/Massachusetts/02/2012 (clade 2) egg-propagated vaccine virus.

Influenza B/Victoria lineage viruses circulating in Georgia during 2015–2016 and 2016–2017 seasons belonged to clade 1A, the B/Brisbane/60/2008 clade. Trivalent influenza vaccines for 2015–2016 contained a virus of the B/Yamagata lineage (a B/Phuket/3073/2013-like virus) while the vast majority of influenza B viruses detected in the European region were B/Victoria lineage [15]. Despite such lineage mismatch, immune responses to the vaccine can provide some cross-protection against infection by viruses of the other influenza B lineage [37]. Genetic studies of the B/Victoria lineage viruses revealed several substitutions in the HA and NA. Circulating viruses did not share the egg-adaptive substitution N197K in HA1, observed in egg-propagated B/Brisbane/60/2008 vaccine virus, but had the substitutions I117V and N129D in HA1. There were a greater number of amino acid substitutions in the NA but the significance, if any, of these substitutions is unknown.

Our study had several limitations that could influence the results of analyses performed. False negative laboratory results may have been obtained due to the late referral of patients to clinics. Individual questionnaires were filled out during sampling and subsequent data regarding course of disease were not collected so we were unable to assess disease severity characteristics (developing pneumonia, need for ICU, etc.). For the same reason, we did not evaluate chronic conditions among non-fatal ILI/SARI cases to determine risk factors associated with any severe courses of disease.

## Conclusions

Despite limitations, this study contributes to a much better understanding of the epidemiological and virologic characteristics of influenza in Georgia. Influenza virus activity in the country was mainly observed from December through March each season with varying peak weeks and predominant viruses. On average one third of patients with ILI/SARI screened in each season had influenza virus infection. Among ILI cases influenza detection was highest in patients aged 5–14 years while the highest proportions of influenza-confirmed SARI cases were among adults. Persons aged 30–64 years had a higher risk of a fatal outcome. The observed circulation of antigenically and genetically variable viruses in Georgia and selection of A/Georgia/532/2015 as a reference virus by WHO CC, London illustrates the need for routine surveillance to contribute to the work of the WHO Global Influenza Surveillance and Response System.

## Supporting information

S1 Fig Map of Georgia. Sentinel site cities are marked in dark green.
(TIF)

S2 Fig NA gene phylogeny of influenza A(H1N1)pdm09 viruses detected in Georgia during 2014–2015 and 2015–2016 influenza seasons. Virus annotation is as for Fig 4.
(TIF)

S3 Fig NA gene phylogeny of influenza A(H3N2) viruses detected in Georgia during three influenza seasons 2014–2017. Virus annotation is as for Fig 5.
(TIF)

S4 Fig HA gene phylogeny of influenza B/Yamagata lineage viruses detected in Georgia during the 2014–2015 influenza season. Vaccine viruses are indicated in red, viruses from Georgia in the 2014–2015 season are shown in blue. Reference and vaccine viruses against which post-infection ferret antisera were raised for use in HI assays are in bold type. The scale bar represents nucleotide substitutions per site.
(TIF)

S5 Fig NA gene phylogeny of influenza B/Yamagata lineage viruses detected in Georgia during the 2014–2015 influenza season. Virus annotation is as for S4 Fig.
(TIF)

S6 Fig HA gene phylogeny of influenza B/Victoria lineage viruses detected in Georgia during 2015–2016 and 2016–2017 influenza seasons. Vaccine virus is indicated in red; 2015–2016 and 2016–2017 viruses from Georgia in pink and brown respectively. Reference and vaccine viruses against which post-infection ferret antisera were raised for use in HI assays are in bold type. The scale bar represents nucleotide substitutions per site.
(TIF)

S7 Fig NA gene phylogeny of influenza B/Victoria lineage viruses detected in Georgia during 2015–2016 and 2016–2017 influenza seasons. Virus annotation is as for S6 Fig.
(TIF)

S1 Table HA and NA sequence accession numbers for all reference and test influenza viruses. (XLS)

S2 Table Influenza-associated deaths during 2014–2017 influenza seasons, Georgia. (TIF)
