# Supplementary figures and images for: Overview of three influenza seasons in Georgia, 2014–2017
Source: PLoS One. 2018 Jul 27;13(7):e0201207. doi: 10.1371/journal.pone.0201207 (PMC6063423; doi:10.1371/journal.pone.0201207)

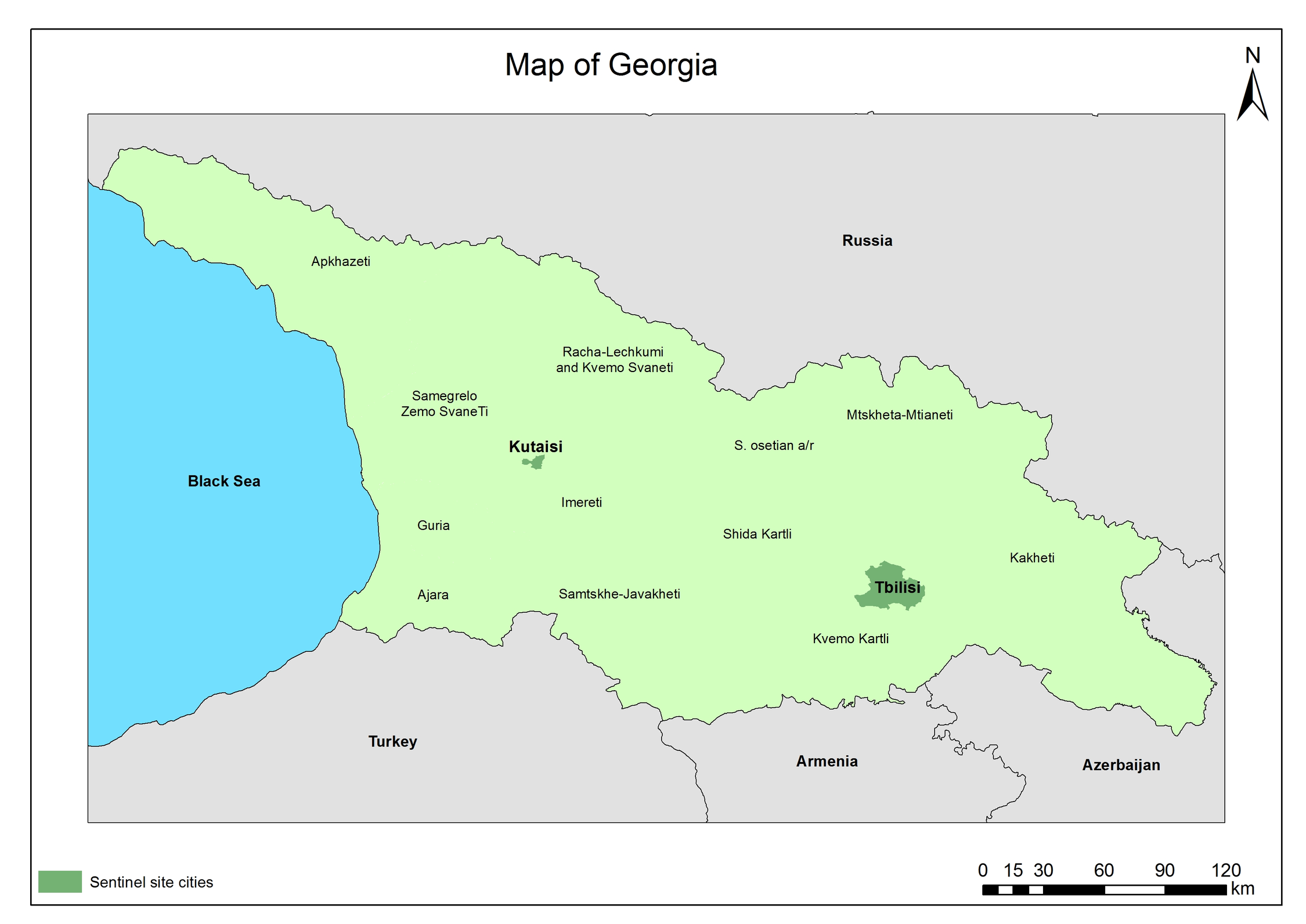

Supplement: S1 Fig — Sentinel site cities are marked in dark green. (TIF) [file pone.0201207.s001.tif]

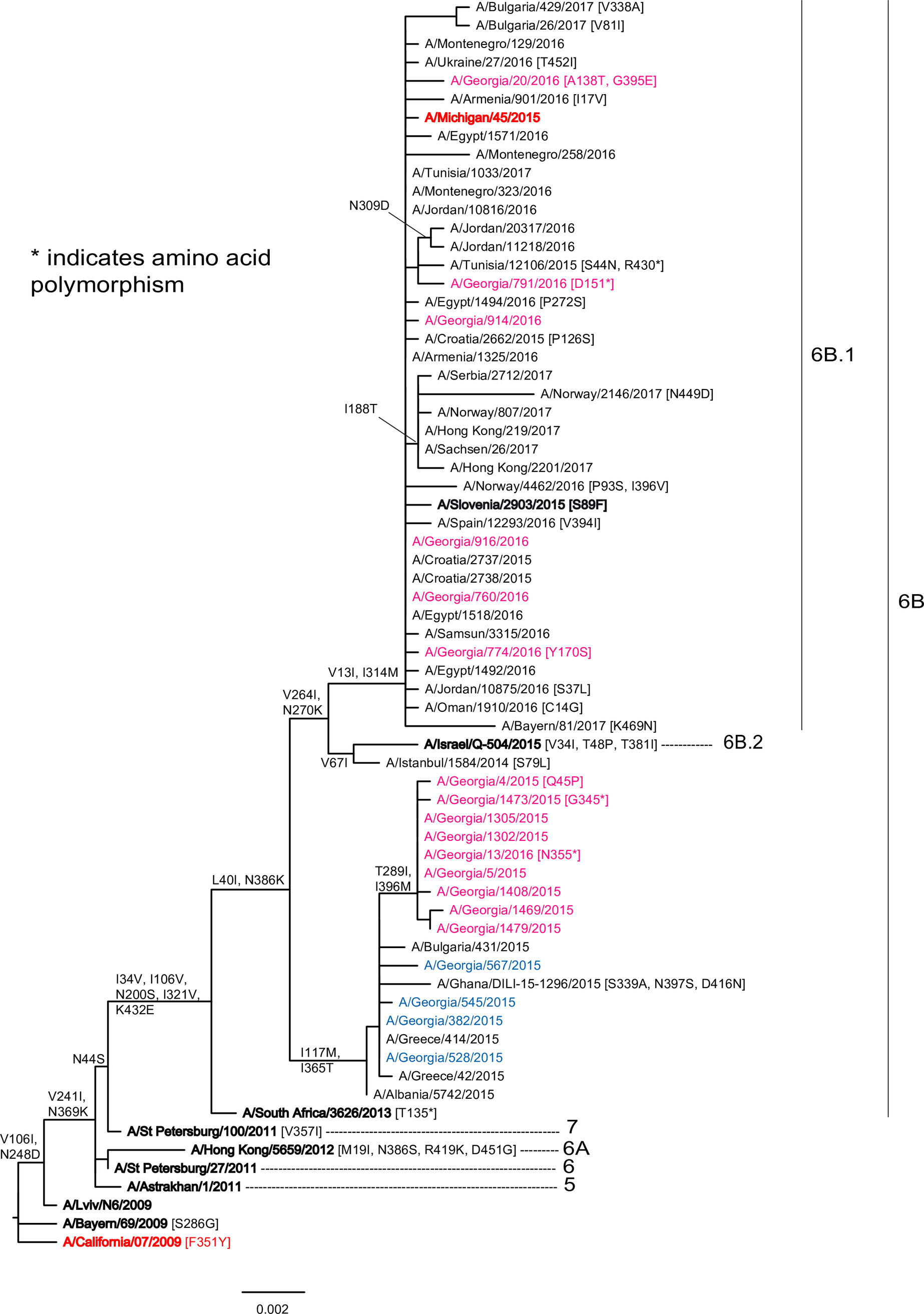

Supplement: S2 Fig — Virus annotation is as for Fig 4. (TIF) [file pone.0201207.s002.tif]

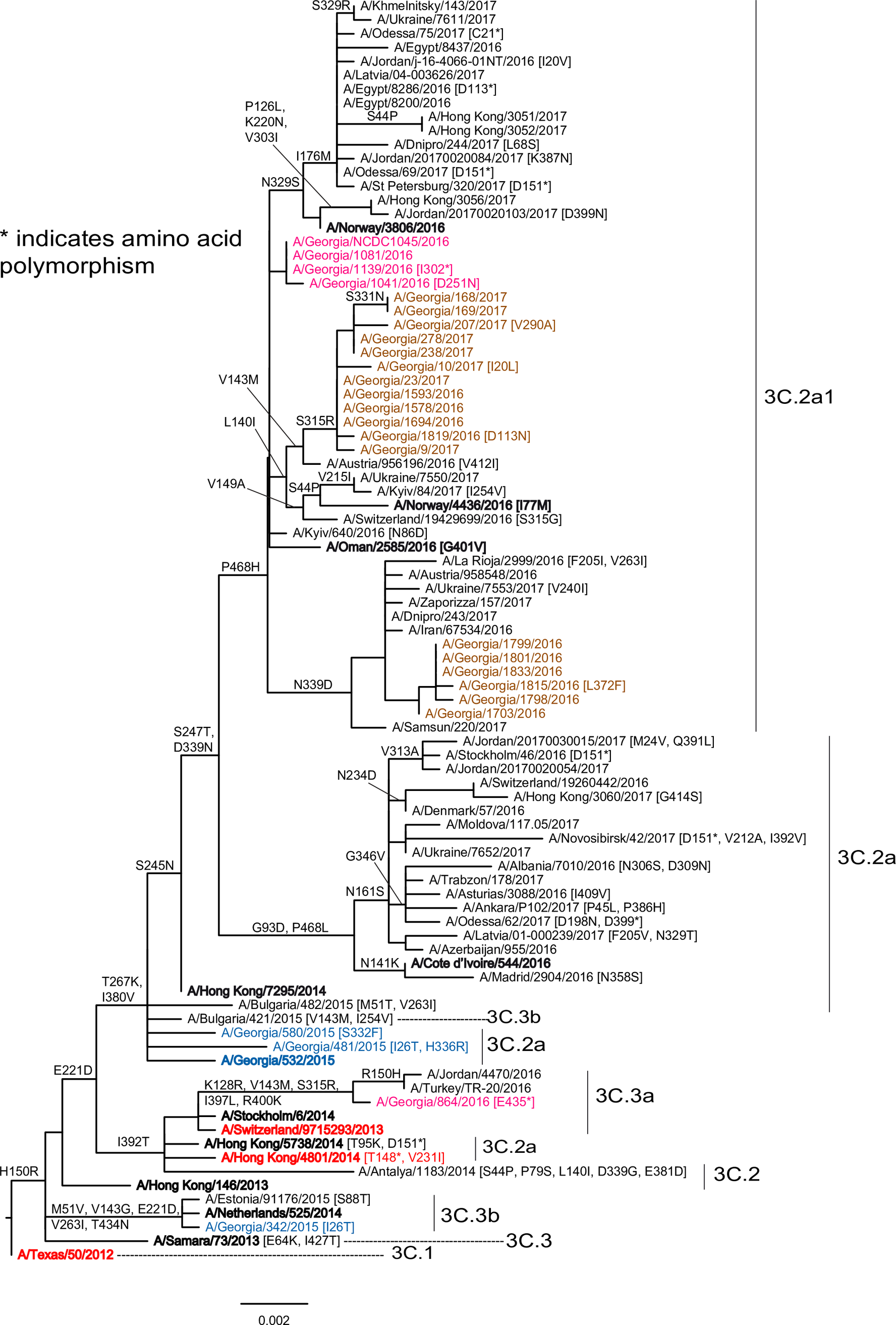

Supplement: S3 Fig — Virus annotation is as for Fig 5. (TIF) [file pone.0201207.s003.tif]

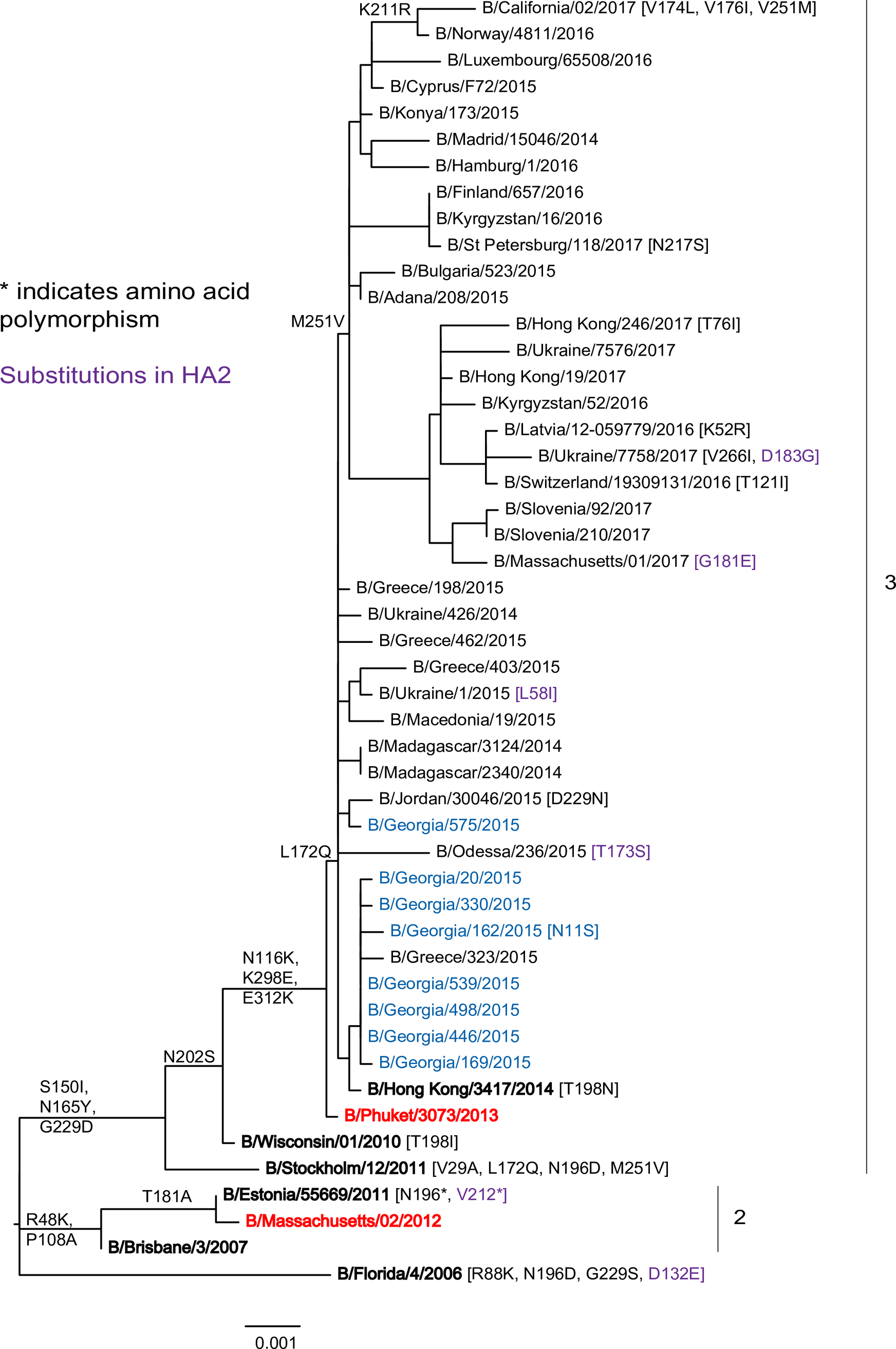

Supplement: S4 Fig — Vaccine viruses are indicated in red, viruses from Georgia in the 2014–2015 season are shown in blue. Reference and vaccine viruses against which post-infection ferret antisera were raised for use in HI assays are in bold type. The scale bar represents nucleotide substitutions per site. (TIF) [file pone.0201207.s004.tif]

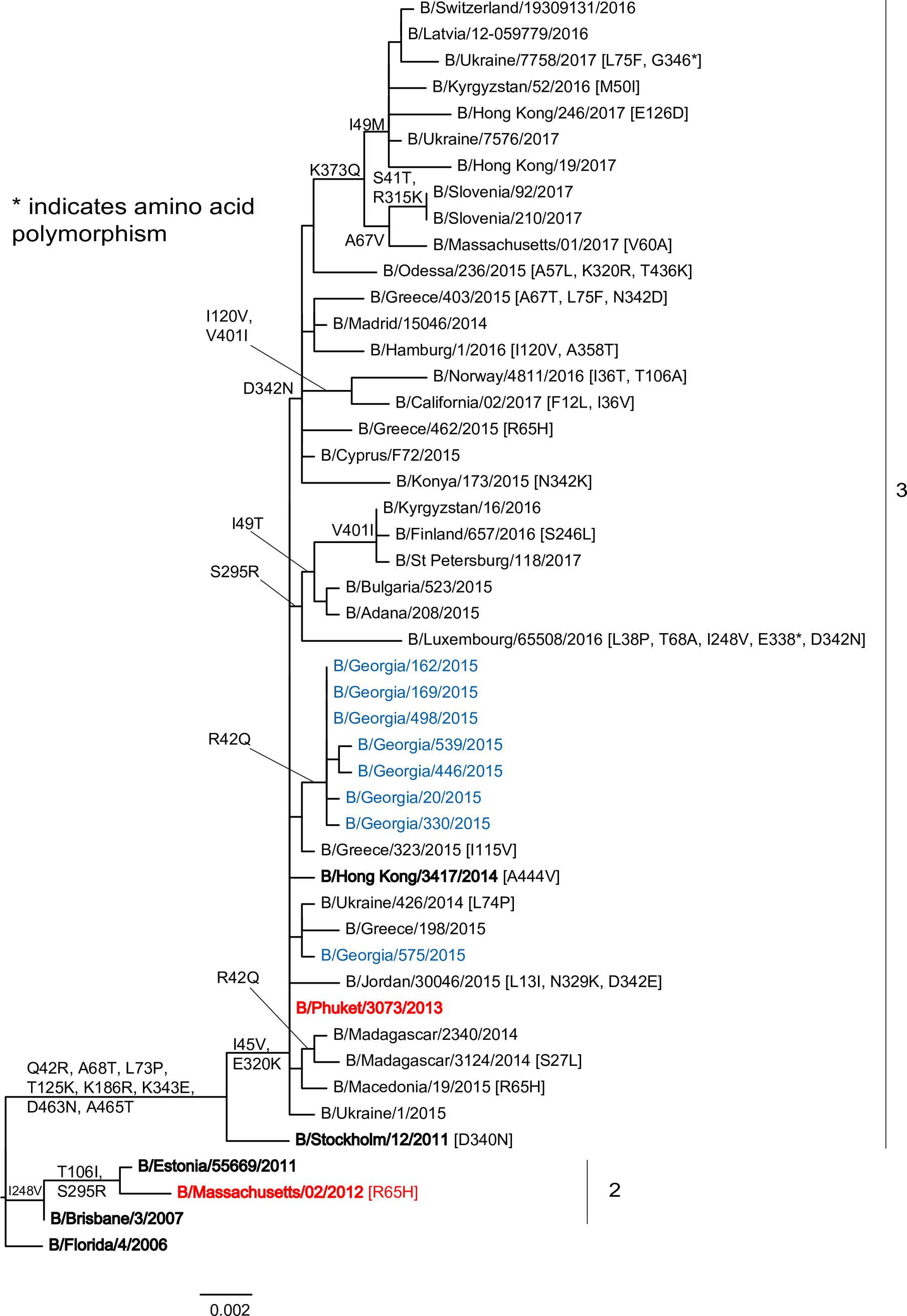

Supplement: S5 Fig — Virus annotation is as for S4 Fig. (TIF) [file pone.0201207.s005.tif]

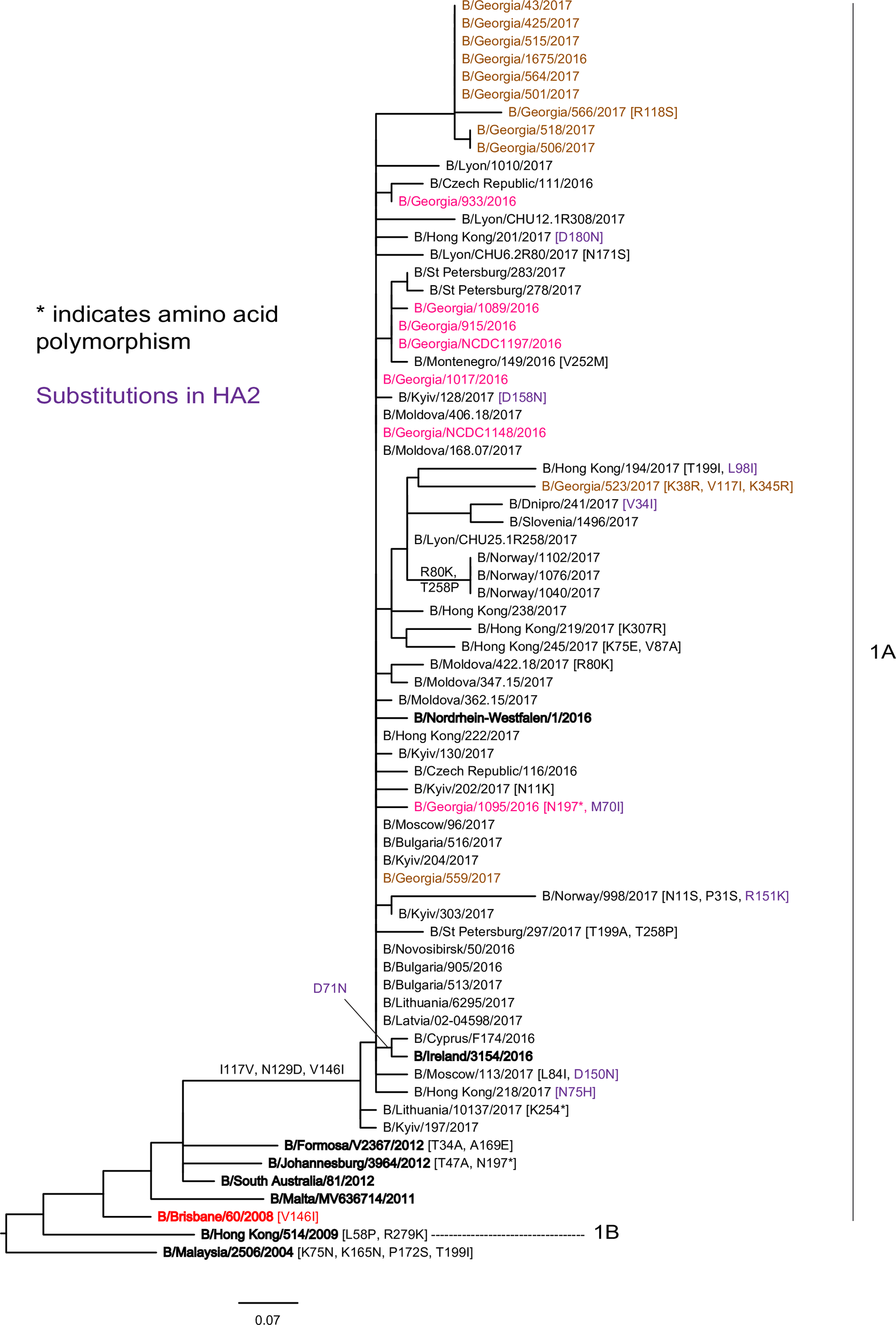

Supplement: S6 Fig — Vaccine virus is indicated in red; 2015–2016 and 2016–2017 viruses from Georgia in pink and brown respectively. Reference and vaccine viruses against which post-infection ferret antisera were raised for use in HI assays are in bold type. The scale bar represents nucleotide substitutions per site. (TIF) [file pone.0201207.s006.tif]

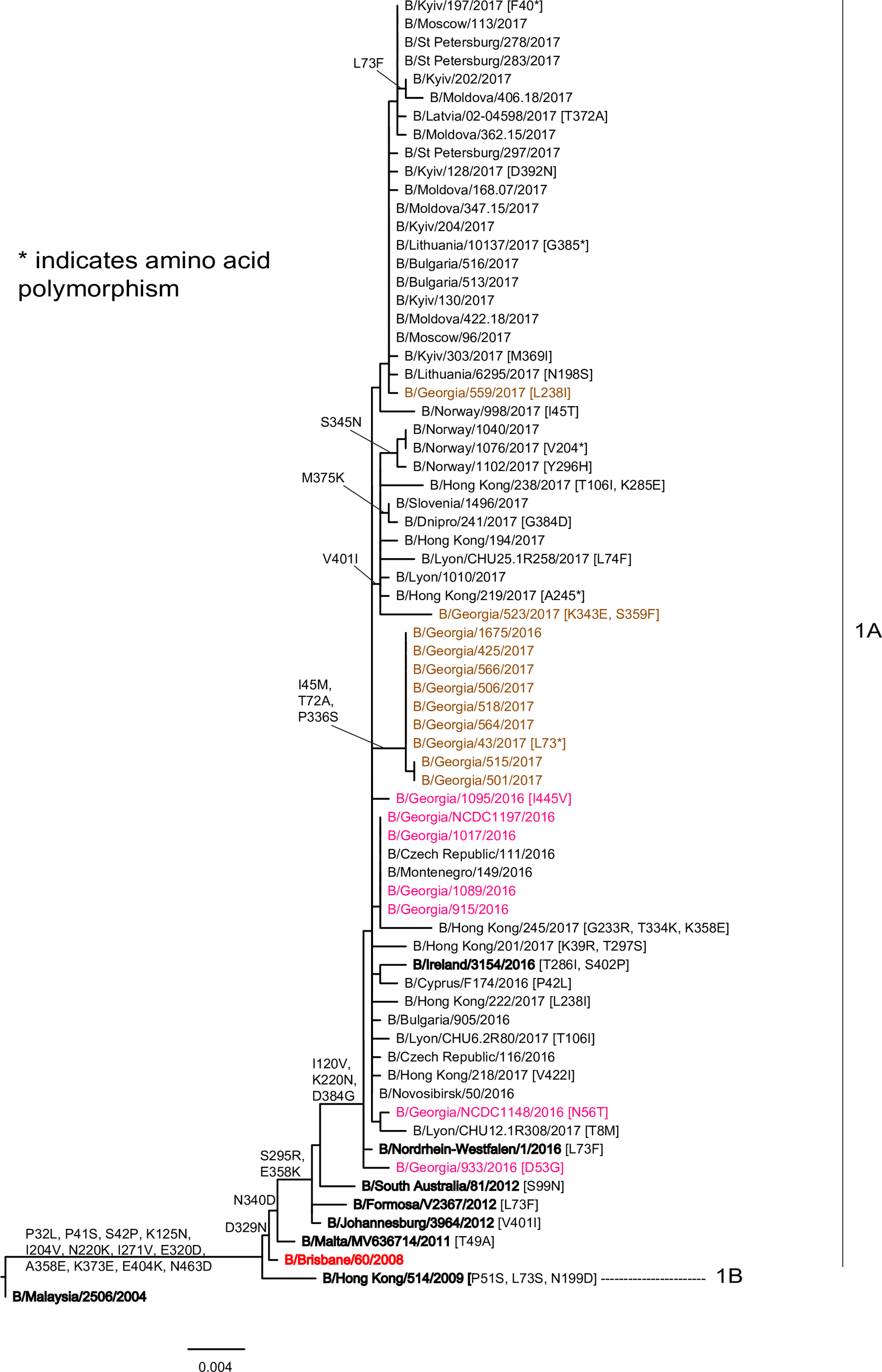

Supplement: S7 Fig — Virus annotation is as for S6 Fig. (TIF) [file pone.0201207.s007.tif]

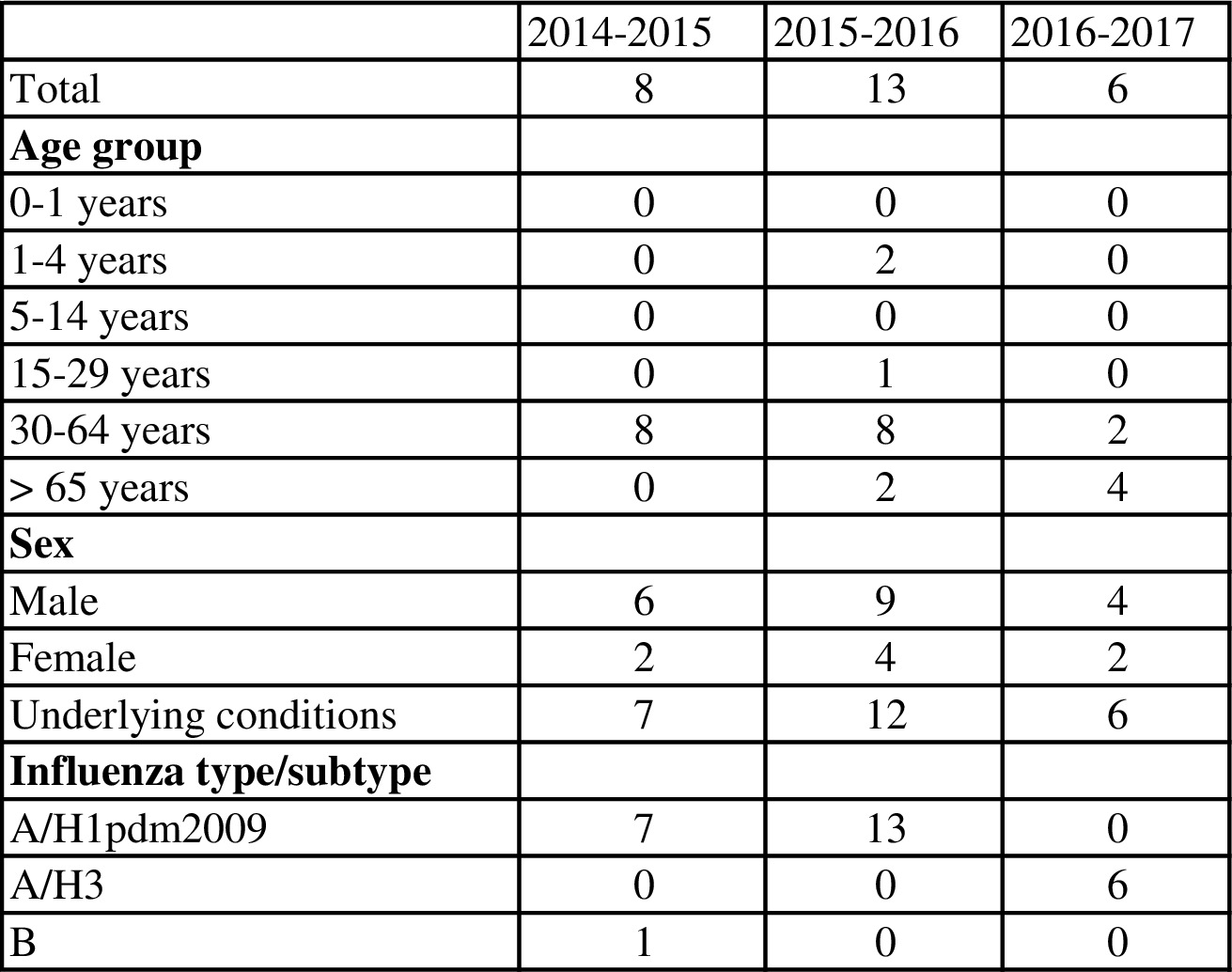

Supplement: S2 Table — (TIF) [file pone.0201207.s009.tif]
